# Single Serum Cortisol Value as a Prognostic Marker in Acute Ischemic Stroke

**DOI:** 10.7759/cureus.40887

**Published:** 2023-06-24

**Authors:** Gurjeet Saini, Kamaldeep Kaur, Lovleen Bhatia, Rupinderjeet Kaur, Jasvir Singh, Gurpreet Singh

**Affiliations:** 1 Department of Medicine, Government Medical College, Patiala, Patiala, IND; 2 Department of Medicine, Government Medical College and Rajindra Hospital Patiala, Patiala, IND; 3 Department of Biochemistry, Government Medical College, Patiala, Patiala, IND

**Keywords:** stress response, national institutes of health stroke scale, prognostic marker, serum cortisol, acute ischemic stroke

## Abstract

Background: Stroke is a major global burden with significant morbidity, mortality, and long-term disability. Acute ischemic stroke (AIS) is a stressful condition causing stimulation of the hypothalamic-pituitary-adrenal (HPA) axis resulting in numerous endocrinal alterations in the body. We evaluated the serum cortisol as a prognostic marker in AIS.

Methods: This was a prospective observational study comprising 100 cases suffering from AIS, and serum cortisol at the baseline was measured. Severity was evaluated using the National Institutes of Health Stroke Scale (NIHSS) during admission, and functional outcome was assessed at 1, 4, and 24 weeks using a modified Rankins score (mRS). Statistical analysis was performed to find the relationship between serum cortisol and the severity of stroke, outcome, and mortality at 1, 4, and 24 weeks of stroke.

Results: In our study, we found positive correlations between random blood sugar and serum cortisol (r = 0.273, p = 0.006); stroke severity (NIHSS) and serum cortisol (r = 0.785, p < 0.001); stroke outcome (mRS) at 1, 4, and 24 weeks; and serum cortisol (p < 0.001 and r = 0.676, 0.654, 0.650 for all three intervals, respectively). We also found higher serum cortisol among patients who died at 1, 4, and 24 weeks compared to those who survived with a p-value being <0.001 for all three intervals.

Conclusions: A stress response causing an increase in serum cortisol occurs in AIS. This response is detrimental to the patient. The serum cortisol at baseline can be considered a marker of severity, short- and long-term prognosis, and mortality after AIS.

## Introduction

According to World Health Organization, stroke is defined as a focal disturbance of cerebral function that lasts more than 24 hours or leads to death and is mediated by vascular damage [1]. Acute ischemic stroke (AIS) is mediated by the activation of hypothalamic-pituitary-adrenal (HPA) axis and sympathoadrenal system. This activation leads to increased levels of stress hormones including cortisol [2,3]. The normal diurnal pattern of serum cortisol levels is altered during acute ischemic stroke [4,5]. However, the effect of this stress response whether it is advantageous or detrimental is not well documented. Cortisol increases the availability of all fuel substrates such as increased glucose level and rapid mobilization of amino acids and fats from cells, making them available to various tissues and organs [6]. On the other hand, this response has been associated with ischemic neuronal injury [7], adverse cardiac effects including arrhythmias [8], and increased infections [9] contributing to increased mortality. Thus increase in cortisol levels has been linked to the severity or prognosis of AIS in various studies [10,11]. In this backdrop, we conducted a study to examine if a single serum cortisol value after AIS can be related to its severity, prognosis, and mortality.

## Materials and methods

This was a prospective observational study conducted at Rajindra Hospital Patiala, Punjab, India, equipped with a stroke unit, and the study was approved by the ethical committee of the hospital with the approval number BFUHS/2k21p-TH/14736. One hundred consecutive patients admitted from January 1, 2021, onward were included.

Inclusion criteria

Patients aged above 18 years who were proven to have their first AIS admitted within 48 hours of acute neurological event by clinical and radiological evidence (CT/MRI brain) were included in the study.

Exclusion criteria

Patients under the age of 18; pregnant or lactating women; patients with hepatic or renal failure; patients taking immunosuppressants, steroids, rifampicin, phenytoin, or psychotropic drugs; patients suffering from malignancy, epilepsy, multiple sclerosis, hemorrhagic stroke, sepsis, and shock, or patients who were thrombolyzed were excluded. Patients who had major surgery in the past three weeks were also excluded.

Any patient presenting within 48 hours of acute neurological deficit or altered mental sensorium was admitted. A detailed history was taken, and a thorough physical examination was done on the day of admission. The patients who had satisfied the inclusion criteria were instructed about the study, and proper informed consent was obtained from the patients. After the history, examination, and consent, patients were subjected to non-contrast computed tomography (NCCT) head (GE Medical Systems, Revolution EVO 128 slice multidetector computed tomography [MDCT] machine) for the evidence of infarction. Patients who were strongly suspected to be suffering from AIS, but did not have infarction on first NCCT, were either subjected to a repeat NCCT or MRI brain (Siemens 1.5 T). In acute ischemic stroke patients, venous blood was obtained on the day of admission for determining complete blood counts with a peripheral blood film, random blood sugar, serum electrolytes, and renal and liver function tests. All blood investigations were performed at the biochemistry and pathology lab of the hospital. The National Institutes of Health Stroke Scale (NIHSS) score was calculated for all patients on the day of admission, and patients were stratified as having minor (1-4), moderate (5-15), moderate-severe (16-20), and severe stroke (21-42) [12].

The following morning after admission, a fasting blood sample from an indwelling venous catheter was obtained for the determination of serum cortisol and fasting lipid profile.

Cortisol measurement

The sample was allowed to coagulate, was centrifuged to eliminate fibrin, and was subjected to cortisol assay on VIDAS® family instruments using VIDAS CORTISOL S (CORS) kit. It is a direct automated quantitative test. The assay was based on the principle of enzyme immunoassay, and the endpoint detection was based on the fluorescent method, which is an enzyme-linked fluorescence assay (ELFA). In this technique, the solid phase receptacle (SPR) serves as the solid phase and the pipetting device. The cortisol interacts with the conjugate (cortisol derivative) present in the well for sites on the specific anti-cortisol antibody coated on the SPR interior. The conjugate enzyme catalyzes the substrate (4-methyl-umbelliferone phosphate) mediated via hydrolysis to form the product 4-methyl-umbelliferone, and the fluorescence was measured at 450 nm. The final calculation was made using the calibration curve.

Following this, the patients were followed up with telephonic interviews or outpatient department (OPD) visits at 1-, 4-, and 24-week intervals for determining the functional outcome using modified Rankins score (mRS) and mortality status. The functional outcomes include symptom-free functional level (mRS score: 0); symptoms but able to work (mRS score: 1); standing still and being able to live independently (mRS score: 2); more severe impairment causing dependency but not loss of ambulation without assistance from another person (mRS score: 3); walker being helped by a caregiver representing the loss of ambulation without the assistance of another person and/or loss of ability to perform bodily self-care (mRS score: 4); bedridden needing continuous care (mRS score: 5); and fatal outcome (mRS score: 6).

Statistical analysis

Continuous variables were shown as mean ± SD, and the categorical variables were represented as frequency and percentage. The normality of the data was confirmed by Kolmogorov-Smirnov and Shapiro-Wilk tests. Kruskal-Wallis H test was applied to compare the means between more than two groups. For correlation studies, Pearson’s and Spearman's test was used. A p-value less than 0.05 was considered statistically significant.

## Results

The baseline demographics and clinical characteristics of the study population were shown in Table 1. The mean age of the patients was 59.31 ± 13.75 years with 54 males and 46 females. The major risk factors were deranged lipid profile and hypertension followed by diabetes mellitus, coronary artery disease, smoking, and atrial fibrillation. The most common derangement in lipid profile was found to be low high-density lipoprotein (HDL) followed by high cholesterol and high triglycerides. The mean systolic blood pressure (SBP) and diastolic blood pressure (DBP) of the population were 147.58 ± 24.85 and 86.36 ± 10.05 mmHg, respectively. Among the stroke syndromes, most patients suffered partial anterior circulation syndrome (PACS) followed by lacunar syndrome (LACS), total anterior circulation syndrome (TACS), and posterior circulation syndrome (POCS). After etiological evaluation, large vessel occlusion (LVO) was the culprit in most cases, followed by small vessel occlusion (SVO), cardio-embolism (CE), and other etiology. The etiology remained unknown in 32% of patients. The mean serum cortisol in our population was 550.13 ± 263.37 nmol/L. The mean cortisol values in TACS, LACS, PACS, and POCS were 803.62 ± 91.75, 562.44 ± 275.27, 539.20 ± 255.84, and 359.12 ± 253.19 nmol/L, respectively. The mean serum cortisol level among patients with LVO was highest followed by cardio-embolic stroke, SVO, unknown etiology, and others as shown in Table 1.

**Table 1:** Baseline characteristics of stroke patients LDL: Low-density lipoprotein; VLDL: Very-low-density lipoprotein; HDL: High-density lipoprotein; NIHSS: National Institutes of Health Stroke Scale; RBS: Random blood sugar; TACS: Total anterior circulation syndrome; PACS: Partial anterior circulation syndrome; LACS: Lacunar syndrome; POCS: Posterior circulation syndrome.

Characteristics	Value	
Age in years (Mean ± SD)	59.31 ± 13.75	
Male sex (%)	54 (54%)	
*Vascular risk factors*		
Deranged lipid profile (n=%)	77 (77%)	
Total cholesterol > 200 mg/dL	33 (33%)	
Triglycerides > 161 mg/dL	33 (33%)	
LDL > 153 mg/dL	17 (17%)	
VLDL > 40 mg/dL	15 (15%)	
HDL < 35.3 for male or <42 for females	44 (44%)	
Hypertension	74 (74%)	
Diabetes mellitus	34 (34%)	
Coronary artery disease	23 (23%)	
Smoking	22 (22%)	
Atrial fibrillation valvular non-valvular	9 (9%), 3, 6	
*Clinical findings*		
Systolic blood pressure (mmHg) (Mean ± SD)	147.58 ± 24.85	
Diastolic blood pressure (mmHg) (Mean ± SD)	86.36 ± 10.05	
NIHSS (Mean ± SD)	14.51 ± 7.48	
*Laboratory findings (Mean ± SD)*		
RBS (mg/dL)	181.69 ± 69.60	
Serum cholesterol (mg%)	177.43 ± 50.92	
HDL (mg %)	42.06 ± 10.63	
LDL (mg %)	104.37 ± 45.77	
VLDL (mg %)	30.00 ± 12.57	
Triglycerides (mg%)	149.12 ± 56.83	
Serum cortisol	550.13 ± 263.37	
*Stroke syndrome (n=%)*	*Percentage*	*Cortisol, nmol/L (Mean ± SD)*
TACS	8%	803.62 ± 91.75
PACS	67%	539.20 ± 255.84
LACS	17%	562.44 ± 275.27
POCS	8%	359.12 ± 253.19
*Stroke etiology (n=%)*	*Percentage*	*Cortisol, nmol/L (Mean ± SD)*
Small vessel occlusive	21%	544.81 ± 309.23
Large vessel occlusive	34%	612.97 ± 220.30
Cardio-embolic	9%	570.78 ± 241.56
Other	4%	476.75 ± 280.87
Unknown	32%	490.22 ± 276.75

The severity of the stroke was assessed using NIHSS. The mean NIHSS in our population was 14.51 ± 7.48. As per severity score, five patients had minor, 50 had moderate, 19 had moderate-severe, and 26 had severe strokes. Table 2 shows the association between the severity of stroke and cortisol. Higher mean serum cortisol was found while moving from lower to higher levels of severity. This association was found to be highly significant with a p-value of <0.001. A Spearman’s rank correlation between NIHSS and serum cortisol was obtained with r = 0.785 and p < 0.001 demonstrating a positive correlation between the two.

**Table 2:** Severity of stroke and serum cortisol

Severity of stroke	No. of patients	Serum cortisol (nmol/L; Mean ± SD)	P-value
Minor	5	180.40 ± 145.95	<0.001
Moderate	50	416.00 ± 229.01	
Moderate-severe	19	661.26 ± 187.13	
Severe	26	797.96 ± 81.81	

Table 3 shows the association of serum cortisol with the functional outcome of AIS (mRS). A positive correlation was found between the functional outcome (mRS) and serum cortisol levels at 1, 4, and 24 weeks with an r-value of 0.676, 0.654, and 0.650, respectively. The mean baseline serum cortisol level at all these intervals was higher in groups with poor outcomes.

**Table 3:** Association between stroke outcome and serum cortisol

Weeks	Outcome (mRS)	No.	Serum cortisol (Mean ± SD; nmol/L)	p-value	r-value between mRS and serum cortisol
1	Good (0-2)	14	201.00 ± 181.85	<0.001	0.676
	Moderate (3-4)	37	481.57 ± 187.59		
	Poor (5-6)	49	701.65 ± 212.38		
4	Good (0-2)	24	276.42 ± 198.11	<0.001	0.654
	Moderate (3-4)	32	519.62 ± 201.64		
	Poor (5-6)	44	721.61 ± 191.83		
24	Good (0-2)	26	296.88 ± 212.75	<0.001	0.650
	Moderate (3-4)	32	517.72 ± 193.56		
	Poor (5-6)	42	731.60 ± 190.64		

**Figure 1:**
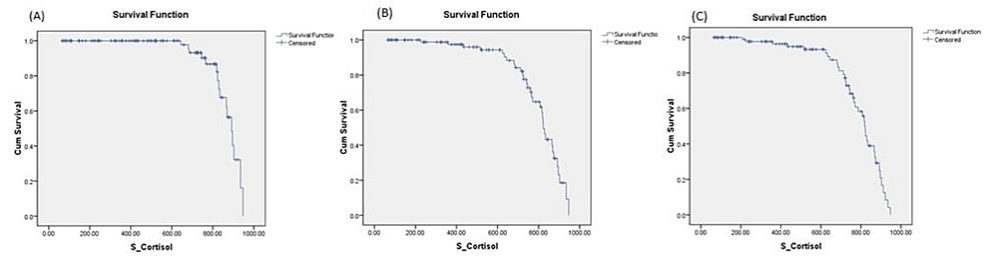
(A-C) Kaplan–Meier survival plots at 1, 4, and 24 weeks

Figure 1 depicts the cumulative survival after AIS as a function of cortisol at 1, 4, and 24 weeks. The plot shows that cumulative survival decreases with higher serum cortisol levels. Table 4 shows that the mean baseline serum cortisol level was significantly higher in non-survivors than survivors at all stages till 24 weeks after AIS, suggesting that higher serum cortisol levels were significantly associated with mortality after AIS.

**Table 4:** Comparison of serum cortisol in dead and alive patients

Weeks	Survival status	Mean ± SD	P-value
1	Dead	820.81 ± 92.48	<0.001
	Alive	498.57 ± 253.70	
4	Dead	740.00 ± 165.55	
	Alive	460.78 ± 253.96	
24	Dead	734.68 ± 180.03	
	alive	437.02 ± 242.27	

The comparison of serum cortisol levels among the dead and alive patients at 1, 4, and 24 weeks were shown in Table 4. At the 1, 4, and 24 weeks, the mean cortisol level was significantly higher in dead patients when compared to alive patients (p < 0.001).

**Figure 2:**
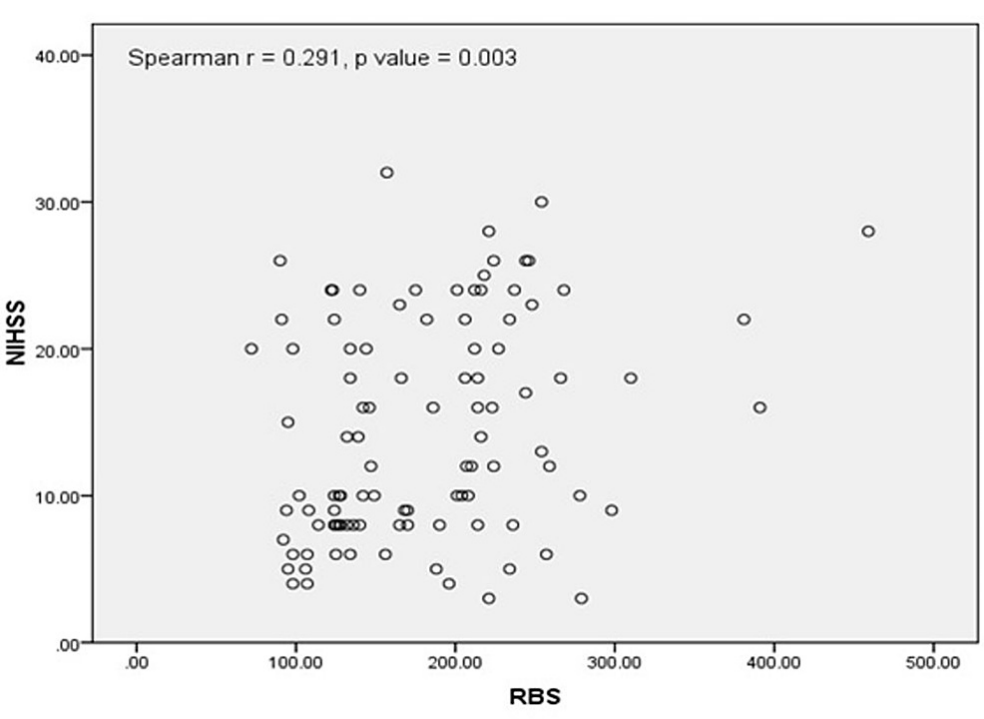
Correlation between RBS and serum cortisol NIHSS: National Institutes of Health Stroke Scale; RBS: Random blood sugar.

Figure 2 shows Spearman’s rank correlation between random blood sugar (RBS) and serum cortisol with r = 0.273 and p = 0.006 demonstrating a positive correlation between RBS and serum cortisol. The mean RBS in our population was 181.69 ± 69.60 mg/dL. The mean RBS among diabetics (n = 34) and non-diabetics (n = 66) was 235.61 ± 58.99 and 129.89 ± 25.77 mg/dL, respectively.

## Discussion

In our study, we found that serum cortisol was positively correlated to RBS, severity of AIS, and functional outcome of AIS at 1, 4, and 24 weeks. Serum cortisol showed a significant association with mortality at 1, 4, and 24 weeks after AIS.

During the follow-up of patients for functional outcome at 1, 4, and 24 weeks using mRS, a positive correlation of outcome was observed with serum cortisol levels on the day following admission at all these intervals and found to be significant (p < 0.001) with Pearson's correlation coefficient value of 0.676, 0.654, and 0.650, respectively. This shows that higher stress response and higher serum cortisol during AIS displayed worsened functional outcomes during short- and long-term follow-up. Various studies in the past correlated serum cortisol levels at baseline to the outcome of AIS at various intervals varying from zero to one year. Zierath et al. reported a significant relationship between serum cortisol and outcome at 1, 3, 6, and 12 months [13]. They found that higher cortisol levels were associated with worse outcomes at these intervals, but the relationship attenuated over time with p-value being <0.001, <0.001, 0.007, and 0.050, respectively, for these intervals. Neidert et al. also concluded that cortisol levels on day 1 after admission mirrored the stroke severity at 90 days and one year [11]. A stroke per se indicates a poor outcome, but the stress response with one of its manifestations as increased cortisol itself leads to worse outcomes mediated by catabolism, increased blood glucose levels, and heart rate. Thus, the increased serum cortisol level showed secondary effects such as neuronal ischemic injury precisely at the hippocampus [7]. A disturbance in the hippocampus function might cause disturbances in the HPA axis as the hippocampus holds a role in feedback regulation of the HPA axis, potentiating cortisol response in addition to the physiological stress response. This stress response is also related to adverse cardiac outcomes including arrhythmias or myofibrillar degeneration and immune dysregulation causing an increased incidence of infections resulting in higher morbidity and mortality [8,11]. The adverse cardiac effects also result from simultaneous sympathetic activation due to stress response. Some studies have shown no significant correlation between cortisol and ischemic stroke [5,13,14,15]. However, all of these studies involved a small number of patients, included patients with mild stroke severity only [15], or used sedatives concomitantly (midazolam or fentanyl), which may have caused variation in the results.

Regarding the mortality after stroke at 1, 4, and 24 weeks, we found a significant association between serum cortisol on day one after admission and mortality at these intervals. We found the levels of cortisol at baseline to be higher in the patients who died than those who survived at these intervals with a p-value of <0.001 for all intervals. Fassbender et al. also reported an early and persisting activation of the hypothalamic-pituitary-adrenal axis, and it shows a significant association with disease severity [16]. Marklund et al. found that higher serum cortisol levels on day 1 predicted both 28-day and one-year mortality [17]. Similar observations were also reported in Neidert et al.'s study where the cortisol levels predicted the day 90 and one-year mortality [11]. On day 90 in Zi et al.'s study [10] and day 7 in Agarwal et al.'s study, serum cortisol levels displayed a significant association with mortality in acute stroke patients [18].

In our study, we observed a positive correlation (r = 0.785) between the severity of AIS (NIHSS) and serum cortisol levels. AIS is a stressful event resulting in stimulation of the HPA axis causing increased serum cortisol levels. As the severity of stroke increases, a higher stress response is mounted resulting in an increased value of serum cortisol. Previous studies done by Neidert et al. and Zi et al. also found a positive correlation between NIHSS and serum cortisol, and it was significant (p < 0.0001) [11,10].

A positive correlation between RBS and serum cortisol (r = 0.273, p = 0.006) can be attributed to higher stress response in hyperglycemia as well as the hyperglycemic effect of cortisol and other stress hormones. Other studies have found a similar correlation, including a multivariate analysis by Zi et al.'s study with an odds ratio of 1.33 per unit increase in glucose level (p < 0.0001) [10]. In another study done by Christensen et al., univariate analysis showed a significant association between glucose level and stroke severity with a regression value of 0.22, and it was significant (p = 0.007) [19].

The limitations of the study were less sample size and the lack of estimation of other stress markers such as adrenocorticotropic hormone (ACTH), noradrenaline, adrenaline, and other hormones involved in the stress response.

## Conclusions

A stress response during AIS showed increased serum cortisol levels. This response is positively correlated to RBS, the severity of AIS as assessed using NIHSS, and the outcome of AIS at 1, 4, and 24 weeks as assessed using mRS, and it also showed a significant association with mortality. Therefore, serum cortisol values could be considered as a prognostic indicator of severity, outcomes, and mortality at intervals of 1, 4, and 24 weeks. This could be due to higher stress response in AIS causing a higher amount of stress hormones including cortisol and activation of the sympathetic system. As discussed, this has been associated with various deleterious effects that contribute to higher morbidity and mortality. Thus, the serum cortisol at baseline can be considered a marker of severity, mortality, and short- and long-term prognosis after AIS.
